# Identification of Potential Pancreatic Lipase Inhibitors from Traditional Chinese Medicines via Molecular Docking, Molecular Dynamics Simulation and In Vitro Validation

**DOI:** 10.3390/cimb48040404

**Published:** 2026-04-15

**Authors:** Zixuan Zhang, Jinhua Long, Tingting Li, Nan Xu, Zhili Xu, Yuedan Wang, Ming Chu, Mingbo Zhang

**Affiliations:** 1 College of Pharmacy, Liaoning University of Traditional Chinese Medicine, Dalian 116600, China; zixuandashen@163.com (Z.Z.); 19071450664@163.com (J.L.); tinng_li@163.com (T.L.); xu-danbs@163.com (N.X.); xu_zhili@126.com (Z.X.); 2 Department of Immunology, School of Basic Medical Sciences, Peking University, NHC Key Laboratory of Medical Immunology (Peking University), Beijing 100191, China; wangyuedan@bjmu.edu.cn

**Keywords:** pancreatic lipase, obesity, traditional Chinese medicine, molecular docking, molecular dynamics simulation, MM/PBSA, inhibitor

## Abstract

Obesity represents one of the most critical global public health challenges. Pancreatic lipase (PL) serves as a key therapeutic target for obesity control, whereas clinical synthetic PL inhibitors are greatly restricted by adverse reactions. Traditional Chinese medicines (TCMs) have a long-standing history in regulating lipid metabolism and ameliorating obesity-related disorders, and are characterized by remarkable structural diversity, low toxicity, and mild side effects, thus representing a promising source for developing safe and efficient PL inhibitors. In this work, an integrated strategy combining in silico screening and in vitro validation was employed to identify potential PL inhibitors from TCM components, including molecular docking, molecular dynamics simulation, MM/PBSA binding free energy computation, and in vitro enzymatic assay. Six compounds with docking scores ranging from −9.9 to −9.0 kcal/mol were selected for further investigation. Molecular dynamics simulations verified the favorable structural stability of the corresponding ligand–PL complexes, and MM/PBSA calculations demonstrated negative binding free energies from −21.24 ± 0.39 to −12.03 ± 0.40 kcal/mol. In vitro experiments indicated that three compounds (Hydroxygenkwanin, Atractylenolide I, and Peiminine) showed effective PL inhibitory activity, with IC_50_ values of 0.128 ± 0.009, 0.584 ± 0.031, and 0.748 ± 0.042 mM, respectively. These values are comparable to quercetin (0.231 ± 0.034 mM) but significantly higher than orlistat (0.481 ± 0.023 μM), which is attributed to their non-covalent binding pattern. Collectively, this study validated the reliability of the integrated in silico and in vitro screening strategy, identified three effective pancreatic lipase inhibitors derived from TCMs, established a robust paradigm for the discovery of natural PL inhibitors, and laid a solid foundation for subsequent research on natural anti-obesity agents.

## 1. Introduction

Obesity is a chronic metabolic disease characterized by the abnormal or excessive accumulation of body fat, which poses a significant threat to human health [1]. The pathogenesis of obesity involves complex interactions among multiple factors. Specifically, genetic predisposition serves as the inherent basis of obesity, with more than 1000 independent genetic variations identified to be associated with this disease [2]. Neurobiological regulation depends on the coordinated interaction between the nervous and endocrine systems; for instance, the hypothalamic arcuate nucleus controls appetite through the release of neuropeptides, and dysregulation of these signaling pathways is closely linked to the pathogenesis of obesity [3]. Unhealthy dietary patterns and increasingly sedentary lifestyles also contribute to the development of obesity [4]. In 2022, over 1 billion people (accounting for 13% of the global population) were obese. It is projected that 1.9 billion adults will be obese by 2035, pushing the global financial burden of obesity to an annual $4.32 trillion by that time [5]. Obesity not only impairs daily activities but also increases the risk of numerous serious diseases, such as stroke, diabetes, atherosclerosis, and cardiovascular diseases [5,6]. Therefore, the prevention and management of obesity bear great medical, social, and economic significance, and the development of safe and effective therapeutic strategies is an urgent clinical demand.

Pancreatic lipase (PL) is the key rate-limiting enzyme responsible for the hydrolysis and absorption of dietary triglycerides in the small intestine. Inhibition of its activity can effectively reduce fat absorption, making it an important therapeutic target for obesity management [7]. Orlistat, a synthetic irreversible covalent pancreatic lipase (PL) inhibitor, is the only clinically approved oral anti-obesity drug targeting PL. However, its clinical use is limited by dose-dependent gastrointestinal side effects such as steatorrhea, flatulence, and rectal pain [8]. Therefore, it is imperative to develop new, safe, and effective PL inhibitors.

Traditional chinese medicines (TCMs) are a complex system of traditional healing that employs natural resources (plants, animals, and minerals) to prevent and treat disease, with a history of thousands of years. The clinical application of TCMs in regulating lipid metabolism and treating “dampness-heat”, “obesity” and other metabolic disorders were recorded in classic TCM works [9]. Many plants derived from traditional Chinese medicine (TCMs) or their formulations, such as *Crataegus pinnatifida*, *Nelumbo nucifera* (leaf), *Panax ginseng*, and *Cassia obtusifolia*, have been demonstrated to possess anti-obesity effects [10,11,12]. A growing number of studies have identified natural products with PL inhibitory activity, such as flavonoids (luteolin, apigenin), triterpenoids (oleanolic acid) and phenolic acids (chlorogenic acid) [13], laying a solid foundation for the systematic screening of PL inhibitors from TCMs. Pharmacological studies have demonstrated that TCMs exert anti-obesity effects via multiple pathways and targets, including regulating pancreatic lipase (PL) activity, inhibiting adipocyte differentiation, improving intestinal microecology, and reducing systemic inflammation [14,15,16]. Natural products derived from TCMs possess the inherent advantages of low toxicity, diverse chemical structures, mild side effects and multi-target synergistic action [17,18], and thus hold great promise as a valuable resource for the development of novel PL inhibitors.

Traditional experimental drug screening methods suffer from several drawbacks, including intensive time and cost requirements, low screening efficiency and difficulty in large-scale compound library screening. Molecular docking, as a structure-based drug discovery technique, can rapidly predict the binding mode and affinity between small molecules and target proteins, thus enabling high-throughput screening of potential bioactive compounds from large compound libraries [19]. However, traditional molecular docking methods have inherent limitations, such as neglect of solvation effects, imperfect scoring functions, and the use of rigid protein conformations that cannot reflect the dynamic changes in the protein–ligand complex [20]. To compensate for these deficiencies, a variety of improved strategies and complementary technologies have been developed: flexible docking or induced-fit docking approaches can partially address the receptor flexibility issue by allowing the movement of key amino acid residues in the active site [21,22]; multiple search algorithms and scoring functions can be used in combination to reduce the bias of a single algorithm [23]. Certainly, it should be noted that all these improvements are achieved at the expense of additional computational power, as the increased complexity of docking strategies requires more intensive computational resources.

Molecular dynamics (MD) simulation can incorporate protein flexibility, mimicking the behavior of the ligand–protein complex in the real biochemical environments [24]. Nevertheless, high computational cost is required, which is particularly notable when dealing with large biomolecular systems or long-time scale simulations [25,26]. Furthermore, binding free energy calculations with methods like Molecular mechanics/Poisson-Boltzmann surface area (MM/PBSA) can provide more accurate ligand–protein binding energy than docking score [27]. Several crude approximations, such as the frequent neglect of conformational entropy and incomplete description of water molecule effects in the binding site, make its performance varied significantly with the tested system [28,29]. In addition, alternative approaches such as thermodynamic integration (TI) and free energy perturbation (FEP)and MM/3D-RISM-KH have shown superior performance in specific scenarios [30,31]. Despite these limitations, integrating molecular docking, MD simulation and MM/PBSA can still effectively improve the efficiency and accuracy of drug screening by complementing each other’s advantages [32,33].

In this study, we systematically screened potential PL inhibitors from TCM constituents through the integration of molecular docking, MD simulations, MM/PBSA binding free energy calculations and in vitro enzymatic validation. This study not only validates the efficiency of the integrated in silico and in vitro screening strategy but also provides valuable reference for the screening and development of novel PL inhibitors.

## 2. Materials and Methods

### 2.1. Screening with Molecular Docking

AutoDock Vina is one of the fastest and most widely used open-source docking engines [34]. Nguyen et al. demonstrated that AutoDock Vina 1.1.2 could redock the co-crystallized PL inhibitor (MUP) into the active site of PL, with an RMSD of 0.86 Å between the docked conformation and the crystal structure (PDB ID: 1LPB), which is below the generally accepted threshold of 2.0 Å [35]. Similar docking protocol was also adopted to screen PL inhibitors from natural products [36,37]. Therefore, the docking parameter and setup validated by Nguyen et al. [35] was employed in the present study, with detailed procedures described below. The PL crystal structure (PDB ID: 1LPB) was pretreated using AutoDock Tools 1.5.6 software [38], including removing water molecules, adding polar hydrogen atoms and assigning atomic charges. The grid box was centered on the centroid of the co-crystallized ligand at (8.4, 24.4, 52.6) Å with box size of 30 × 30 × 30 Å^3^. The molecular structures of all TCM-derived compounds (13,132 in total) were downloaded from the TCMSP database (www.tcmsp-e.com, accessed in 1 March 2022) [39]. SAL-B was manually adjusted to a deprotonated, negatively charged state, while all other molecules remained neutral. The formats of compounds were converted from MOL2 to PDBQT using AutoDock Tools 1.5.6 software.

Compounds exhibiting a docking score lower than −9.0 kcal/mol were selected for subsequent analysis. To ensure structural diversity within the dataset, compounds with a Tanimoto similarity coefficient ≥ 0.6 (calculated based on ECFP4 fingerprints) were clustered into structurally related groups. For each cluster, only the compound with the most favorable docking score was retained. For each selected compound, the conformation corresponding to the lowest binding energy was designated as the optimal docking pose. Molecular visualization of ligand–PL complexes was performed using Discovery Studio Visualizer (BIOVIA Discovery Studio 2019, Dassault Systèmes, San Diego, CA, USA) and Pymol 2.5.5 (Schrödinger, LLC., New York, NY, USA, 2025) [40].

### 2.2. Molecular Dynamics Simulation

Molecular dynamics (MD) simulations were performed using the GROMACS 2024 software package [41]. The structure exhibiting the lowest binding energy from the preceding molecular docking simulation was selected as the initial conformation of the complex for MD simulation. Amber14SB-ILDN all-atom force field [42] and TIP3P solvent model were used to simulate the protein and water molecules respectively. Geometric optimization and electrostatic potential calculations for the ligands were performed using Gaussian 09 [43] software at the HF/6–31G(d) level. The antechamber module implemented in AmberTools23 [44] software package was employed to assign general AMBER force field (GAFF) [45] parameters to the ligands.

Each complex was solvated in an octahedral water box, with the minimum distance from the complex to box boundary set to 1.0 nm. Appropriate numbers of Na^+^ and Cl^−^ ions were added to neutralize the overall charge of system. Before the simulation, energy minimization was performed with the steepest-gradient method to eliminate unreasonable intermolecular interactions in the system. Subsequently, a 300 ps equilibration simulation was performed under the constant temperature and volume (NVT) ensemble at 310 K, followed by a 500 ps simulation under the constant temperature and pressure (NPT) ensemble (310 K, 1 atm) to fully equilibrate the system. Finally, a 100 ns MD production simulation was performed on each system under the NPT condition at a temperature of 310 K and 1 atm pressure with the time step of 2 fs. Subsequent trajectory analysis included the time-dependent variations in root mean square deviation (RMSD), root mean square fluctuation (RMSF), radius of gyration (Rg) and the number of hydrogen bonds. MM/PBSA is a classical molecular mechanics-based approach used to quantify the binding affinity between receptors and ligands [46]. In this study, 80 conformations were extracted at 125 ps intervals from the last 10 ns of the MD trajectory for MM/PBSA calculations.

### 2.3. Materials and Reagents

Porcine pancreatic lipase was purchased from Sigma-Aldrich Co. (St. Louis, MO, USA). Orlistat, p-nitrophenyl palmitate (pNPP), Atractylenolide I, Linarin, Hydroxygenkwanin, Salvianolic acid B, Peiminine and Mulberroside A, Quercetin were all purchased from Yuanye Bio-Technology Co., Ltd. (Shanghai, China). All the chemical reagents used in this study were of analytical grade.

### 2.4. Inhibition Rate and Inhibition Type on Pancreatic Lipase

The experiment was performed according to the method reported by Glisan et al. [47] with minor modifications, and all experiments were carried out in triplicate. Briefly, 20 μL of PL solution (6.5 mg/mL), 10 μL of test compound solution, and 160 μL of PBS buffer (pH 8.0) were mixed and preincubated at 37 °C for 10 min to facilitate sufficient binding between the enzyme and the test compounds. The enzymatic reaction was initiated by the addition of 10 μL pNPP solution (3 mg/mL). Absorbance at 405 nm was recorded at 2 min intervals over a 20 min period. The linear region of the absorbance–time curve was used to determine the reaction rate K (i.e., the slope of the linear regression). The inhibition ratio was calculated using Equation (1):

(1) $$\mathrm{Inhibition}\;\mathrm{rate}\;\left(\%\right)\;=\;\left[1\;-\;\frac{K_{a}-K_{b}}{K_{c}}\right]\;\times \;100\%$$

where K_a_ is the reaction rate with the presence of the test compound, K_b_ is the blank reaction rate without enzyme, and K_c_ is the reaction rate without test compound.

The inhibition type was explored in a way similar to that described above. The reaction rate (*v*) was calculated according to the changes in absorbance over time. The reaction rates were measured under the condition that the concentration of the inhibitor was held constant and the concentration of the pNPP ([S]) was varied. Then the reverse of *v* was fitted with the reverse of [S]. According to Michaelis-Menten equation,

(2) $$\frac{1}{v}=\frac{K_{m}}{V_{\max}}\;\times \;\frac{1}{\left[S\right]}+\frac{1}{V_{\max}}$$

where K_m_ is the Michaelis constant and V_max_ is the maximum reaction rate. Inhibition type of the compounds could be determined by analyzing the slopes, intercepts and intersection points of the fitted curves.

### 2.5. Statistical Analysis

All in vitro experiments were performed in triplicate. Data were expressed as mean ± standard error of the mean (SEM). IC_50_ values were log-transformed before statistical comparison. An unpaired two-tailed *t*-test was used to compare differences between groups, and *p* < 0.05 was considered statistically significant. Curve fitting and plotting were performed using the plotting module of scikit-learn [48], and statistical analyses were carried out using GNU PSPP software (version 2.0.1, https://www.gnu.org/software/pspp/, accessed on 28 December 2025).

## 3. Results

### 3.1. Molecular Docking

**Figure 1:**
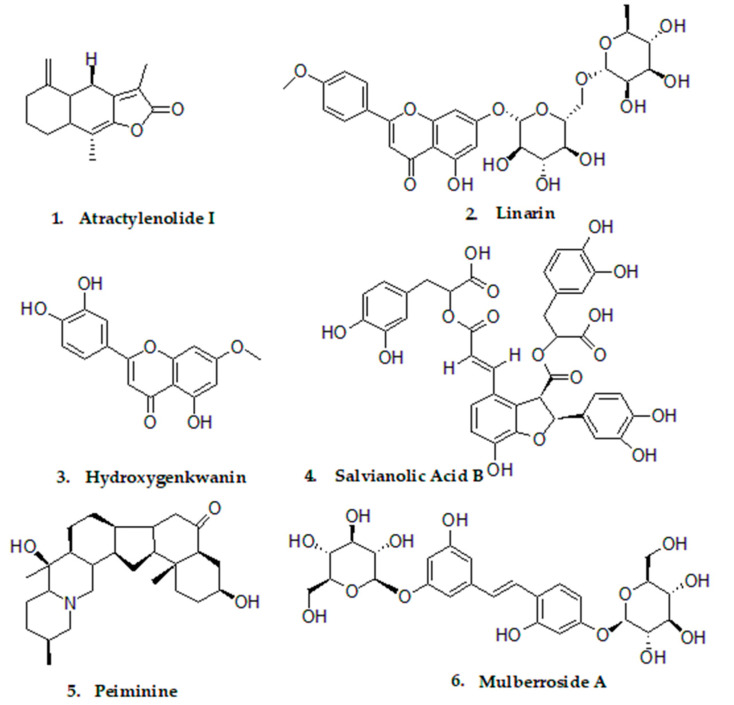
Chemical structure of the selected candidates from molecular docking.

Molecular docking serves as an effective tool for screening large compound libraries and identifying potential bioactive hits [19]. A total of 13,132 compounds from TCMSP were screened with Autodock Vina. Analysis of molecular docking results showed that the docking scores, which reflect the binding energies of the known natural product inhibitors of pancreatic lipase (PL) [13,49,50,51,52], were generally below −7.0 kcal/mol, as summarized in Table S1. To reduce the number of candidates for the subsequent MD simulations, only compounds with binding energies below −9.0 kcal/mol were selected. After removing structurally similar compounds (Tanimoto coefficient ≥ 0.6), six compounds with no previously reported pancreatic lipase (PL) inhibitory activity were selected for further investigation. The similarity matrix among them is presented in Figure S1. The docking scores and key interactions with PL were listed in Table 1. The chemical structures of these selected compounds were shown in Figure 1.

**Figure 2:**
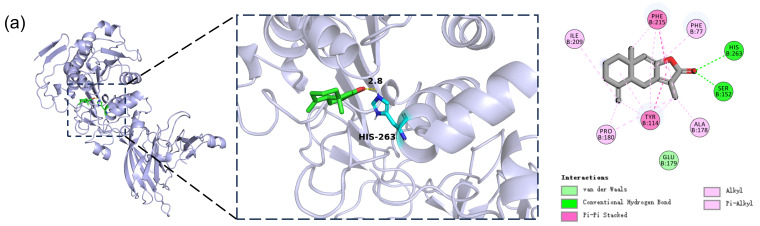

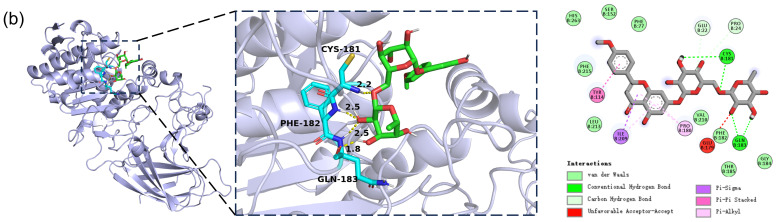

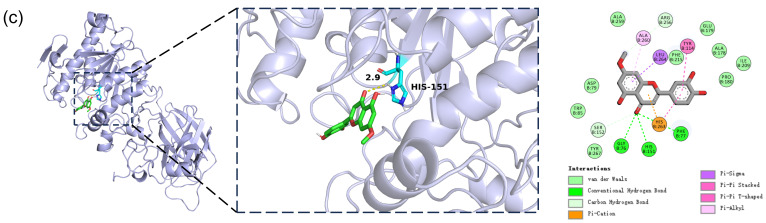

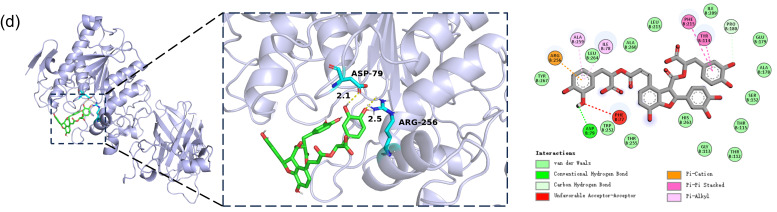

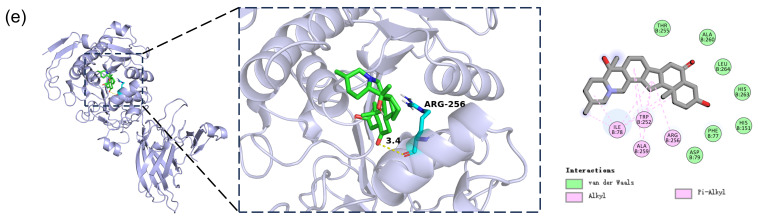

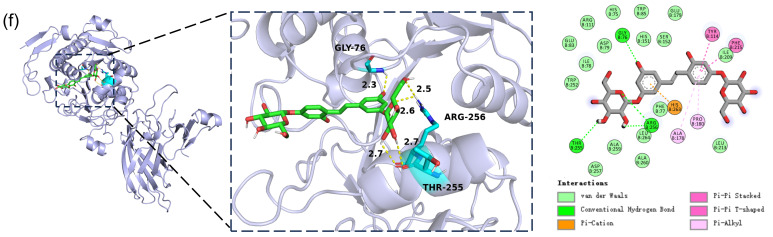
Binding conformations of the compounds with PL. (**a**) ATR-I; (**b**) LIN; (**c**) HYD; (**d**) SAL-B; (**e**) PEI; (**f**) MUL-A.

Hydrophobic interactions have been previously identified as key drivers of inhibitor-PL binding [53]. Binding conformation analysis revealed that all candidate compounds interacted with hydrophobic residues (e.g., Phe77, Tyr114, Phe215), which formed a hydrophobic cleft critical for substrate recognition [54]. Additionally, all compounds except **PEI** formed conventional hydrogen bonds with polar residues like Ser152, His263, Gly76 and His151 (Table 2). Figure 2 showed the detailed binding conformations of the compounds with PL. **ATR-I** established two hydrogen bonds with the Ser152 and His263 residues respectively, through its carbonyl group, along with π–π interactions with Phe215 and Tyr114 (Figure 2a). **LIN** formed four hydrogen bonds with Cys181 and Gln183, one π–π interaction and one π–σ interaction with Tyr114 and Ile209 respectively (Figure 2b). **HYD** binds to the active site of PL via multiple interactions, including three hydrogen bonds with Gly76, Phe77, and His151, a π–π interaction with Tyr114, and a π–σ interaction with Leu164 (Figure 2c). **SAL-B** forms a hydrogen bond with Asp79, two π–π stacking interactions with the side chains of Phe215 and Tyr114, and a π–cation interaction with Arg256 (Figure 2d). **PEI** formed two π–alkyl interactions with Ile78 and Trp252, along with hydrophobic interactions with residues such as Phe79, His151, His263 and so on (Figure 2e). **MUL-A** established multiple conventional hydrogen bonds with residues including Arg256, Thr255, and Gly76, accompanied by multiple π–π and π–σ interactions with residues Tyr114, Phe215, Pro180, and Ala178 (Figure 2f). Although minor unfavorable interactions were observed for **LIN** and **SAL-B**, the overall synergistic effects of multiple non-covalent forces still favorable for the formation of ligand–PL complexes, as indicated by their negative binding energies.

### 3.2. Molecular Dynamics Simulation Analysis

To further evaluate the structural stability of the ligand–PL complexes, molecular dynamics (MD) simulations were carried out using the docked conformations as initial structures. For comparative analysis, MD simulations were also performed on apo PL. The dynamic properties of these systems were characterized by analyzing the root mean square deviation (RMSD), root mean square fluctuation (RMSF), radius of gyration (Rg), and the number of intermolecular hydrogen bonds.

#### 3.2.1. Analysis of the RMSD, RMSF and Rg

**Figure 3:**
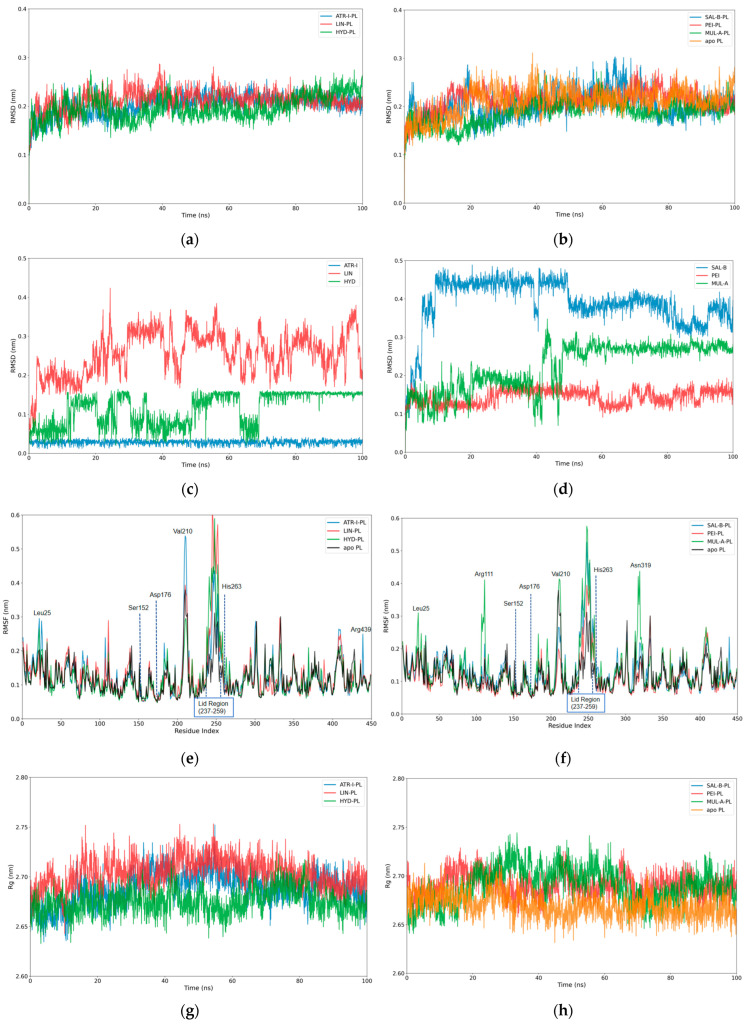
Analysis of molecular dynamics simulations for ligand–PL complexes and apo PL. (**a**,**b**) The backbone RMSD plots of PL in ligand-bound and apo states; (**c**,**d**) the RMSD plots of the compounds; (**e**,**f**) radius of gyration (Rg) plots of ligand–PL complexes compared with the apo PL; (**g**,**h**) the RMSF profiles for ligand–PL complexes and apo PL.

As illustrated in Figure 3a,b, the backbone RMSD of PL remained below ~0.25 nm for most of the simulation time across all systems. This result indicates that the structure of PL, whether in the ligand-bound or apo state, exhibited high stability under the simulation conditions employed. In contrast, the RMSD of the six binding compounds varied significantly (Figure 3c,d). The RMSD of **ATR-I** (blue curve in Figure 3c) displayed only minor fluctuations within 0.05 nm throughout the entire simulation, owing to its rigid structure. **LIN** (red curve in Figure 3c) displayed the most pronounced and frequent fluctuations among all compounds, with a mean RMSD value of 0.26 nm, indicating substantial conformational variability and an unstable binding state. **HYD** (green curve in Figure 3c) exhibits moderate fluctuations during the initial simulation phase, followed by a stabilization at a lower RMSD level (~0.15 nm) with only minor oscillations after approximately 70 ns, suggesting a gradual convergence toward a stable binding conformation. The RMSDs of **PEI** (red curve in Figure 3d) kept below 0.2 nm throughout the simulation, and showed minor fluctuations in the last 10 nm, indicating a relative stable binding conformation. **MUL-A** exhibited several distinct RMSD fluctuations (green curve in Figure 3d), with peaks exceeding 0.3 nm during the 40–60 ns interval, then gradually stabilized to a plateau over the final 30 ns of the simulation, indicating that its binding conformations likely evolved toward more stable states. As shown in Figure 3d (blue curve), **SAL-B** displayed the highest RMSD values throughout the simulation, with a mean RMSD of 0.39 nm. Its RMSD profile exhibited a continuous increase during the first 60 ns, plateauing at approximately 0.32 nm, and then fluctuated stably between 0.30 and 0.40 nm until the end of the 100 ns simulation. The relatively high RMSD values of **SAL-B** suggested that it induces substantial conformational rearrangements within the binding pocket of PL, leading to comparatively lower binding stability.

The root mean square fluctuation (RMSF) analysis was performed to evaluate the dynamic flexibility of residues of PL in both apo and ligand-bound states (Figure 3e,f). It was observed that the majority of residues exhibit RMSF values below 0.20 nm, indicating a stable overall structural framework across all systems. In particular, the residues around the key residues responsible for the catalytic activity of PL (i.e., Ser152, Asp176 and His263) showed RMSF values below 0.1 nm, indicating that binding of the ligand stabilizes the catalytic domain of PL. In comparison to the residues in apo PL, the residues in ligand–PL complexes showed notable peaks of high flexibility in the lid region (residues 237–259), which controls access to the catalytic pocket. This enhanced flexibility reflected a ligand-induced transformation of the lid into a persistently open conformation, which prevented the enzyme from adopting the closed, catalytically active state required for substrate binding and hydrolysis. In addition, the **ATR-I-PL** complex displays pronounced peaks at residues Leu25, Val210 and Arg429 (blue curve in Figure 3e), while **MUL-A-PL** exhibits prominent flexibility peaks at residues Leu24, Arg110, Val210, and Asn319, indicating significant conformational rearrangements induced by ligand binding (green curve in Figure 3f).

The radius of gyration (Rg) is usually used to assess the structural compactness of proteins. A smaller Rg value indicates a more compact and stable protein conformation. The Rg of the apo-form of PL and the six ligand–PL complexes were analyzed, with the resulting plots presented in Figure 3e,f. The apo PL system (orange curve in Figure 3f) exhibited fluctuations within a narrow range of 2.65–2.70 nm and displayed a gradual decreasing trend over the course of the simulation, suggesting enhanced structural stability as the system approached equilibrium. The Rg values of all complexes ranged from 2.65 to 2.75 nm and exhibited a narrow fluctuation range. Collectively, these results indicated that PL maintained structural stability in all systems throughout the simulation, with ligand binding causing negligible disturbance to its global structure.

#### 3.2.2. Variation in Binding Modes During the Simulation

**Figure 4:**
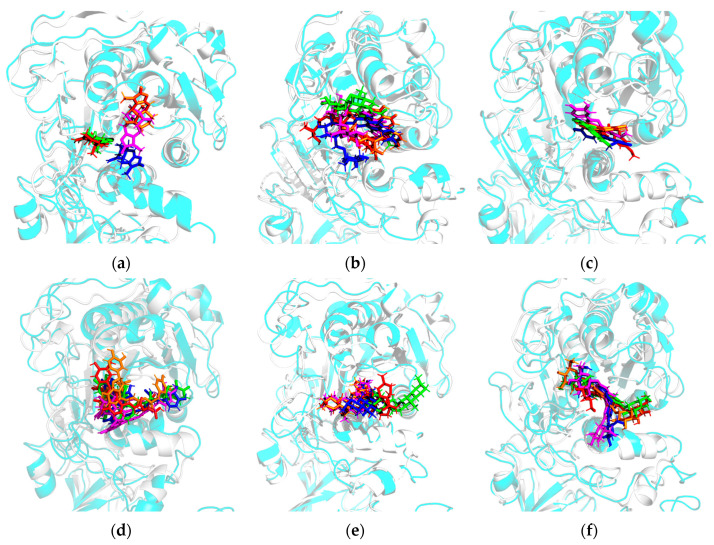
The variation in binding mode of compounds after aligning the PL backbone, at 0 (red), 25 (green), 50 (orange), 75 (megenta), and 100 (blue) ns. (**a**) ATR-I; (**b**) LIN; (**c**) HYD; (**d**) SAL-B; (**e**) PEI; (**f**) MUL-A. The cartoon representations of PL conformations at 0 ns and 100 ns are colored in gray and cyan, respectively.

To better characterize the dynamic binding behavior of the ligands, the positions and conformations of each compound at representative time points (0, 25, 50, 75, and 100 ns, colored red, green, orange, megenta, and blue, respectively) were visualized after superposition of the PL backbone (Figure 4). **ATR-I** departed from its initial binding site at 50 ns (Figure 4a), then partially reverted to its original position and adopted an optimized binding conformation. **LIN** and **SAL-B** exhibited obvious variations in their positions and orientations throughout the simulation, accompanied by substantial conformational adaptation (Figure 4b,d). These observations are consistent with their large mean RMSD values, indicating an unstable binding state. **HYD** maintained a relatively stable binding conformation throughout the simulation. Its core structure remained anchored within the active site of PL, with only minor adjustments observed in the orientation (Figure 4c). **PEI** exhibited substantial conformational fluctuations over the course of the simulation, indicative of its inherent flexibility, yet underwent no significant spatial displacement within the binding site (Figure 4e). **MUL-A** stayed within the original binding pocket, optimizing its binding interactions with PL with merely minor conformational changes (Figure 4f).

#### 3.2.3. Hydrogen Bond Analysis

**Figure 5:**
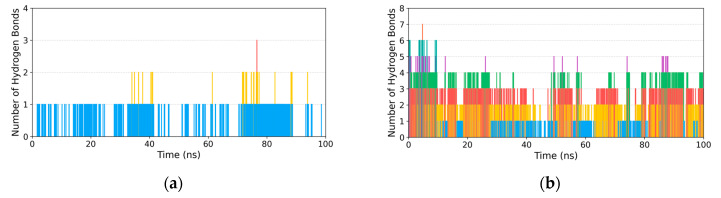

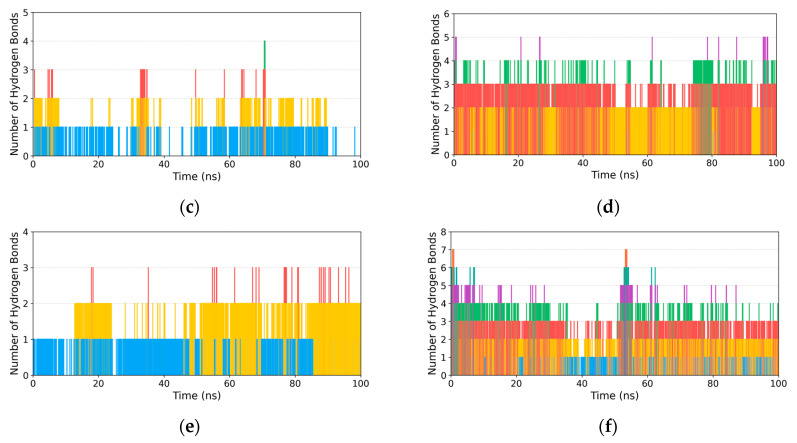
Time-dependent hydrogen bond profiles between the compounds and PL. (**a**) ATR-I; (**b**) LIN; (**c**) HYD; (**d**) SAL-B; (**e**) PEI; (**f**) MUL-A.

Hydrogen bonds are crucial non-covalent interactions that contribute significantly to the binding affinity. Figure 5 showed the number of hydrogen bonds formed between the compounds and PL throughout the MD simulations. For **ATR-I**, **HYD** and **PEI** (Figure 5a, Figure 5c and Figure 5e, respectively), the number of hydrogen bonds generally are no more than three, with only one hydrogen bond observed in the majority of cases. For **LIN**, **SAL-B**, and **MUL-A** (Figure 5b,d,f), the number of hydrogen bonds remained above three for most of the simulation time, indicative of a strong and dynamic hydrogen-bond network with PL arising from the multiple hydroxyl groups present in these compounds.

#### 3.2.4. MM/PBSA Calculation

The MM/PBSA method is a computational technique for evaluating the binding affinity of ligands with protein [27]. In this study, the binding free energies between the six compounds and the PL were calculated using the MM/PBSA method. The detailed energy components are summarized in Table 2. All tested compounds exhibited negative total binding free energies (ΔG_TOTAL_), suggesting spontaneous and stable binding with the protein. Among them, **PEI** showed the most favorable binding affinity with a ΔG_TOTAL_ value of −21.24 ± 0.39 kcal/mol, followed by **LIN** (−19.54 ± 0.43 kcal/mol) and **HYD** (−15.58 ± 0.85 kcal/mol). **ATR-I** and **MUL-A** showed similar binding affinity with ΔG_TOTAL_ of −13.44 ± 0.45 and −13.33 ± 0.58 respectively. In contrast, **SAL-B** displayed the highest ΔG_TOTAL_ (−12.03 ± 0.40 kcal/mol), indicating its relatively weak binding stability, which was consistent with the higher RMSD values observed in the molecular dynamics simulations.

Further energy decomposition revealed that van der Waals interactions (ΔE_VDW_) contributed favorably to the binding of all compounds, reflecting the importance of hydrophobic packing and shape complementarity between the ligands and the protein binding pocket. For most compounds except **SAL-B**, electrostatic interactions (ΔE_ELE_) were also energetically favorable, yet were partially counterbalanced by polar solvation effects (ΔE_PB_). Nonpolar solvation energies (ΔE_NPOLAR_) were energetically beneficial for all investigated compounds, with comparable magnitudes across the series. As indicated by the negative values of ΔG_GAS_, the binding of these compounds to PL was driven by direct protein–ligand interactions. In contrast, **SAL-B** exhibited a distinctly abnormal energy profile. It exhibited a strongly positive electrostatic energy (ΔE_ELE_ = 75.58 ± 2.13 kcal/mol). This can be attributed to the presence of multiple negatively charged residues in the active site of pancreatic lipase (PL), such as Asp176 and Leu153. Molecular docking revealed that other negatively charged residues, including Glu179, Leu213, Leu264, and Asp179, are also distributed around the binding pocket of **SAL-B** (Figure 2d). In addition, SAL-B possesses a high hydration energy (−59.06 ± 1.88 kcal/mol) in aqueous solution due to its charged nature, which also disfavors its binding to PL. Consequently, the gas-phase binding energy (ΔG_GAS_) of **SAL-B** was positive, whereas its solvated binding energy (ΔG_SOLV_) was −62.70 ± 1.87 kcal/mol, implying that its binding was predominantly driven by solvation effects rather than direct protein–ligand interactions.

Overall, all compounds exhibited negative binding free energies with PL, indicating that the formation of ligand–PL complexes was thermodynamically favorable. Although MM/PBSA generally provides more reliable results than docking methods, its performance in ligand ranking is still unsatisfactory [27]. Therefore, all six compounds were selected for subsequent in vitro validation.

### 3.3. In Vitro Validation

**Figure 6:**
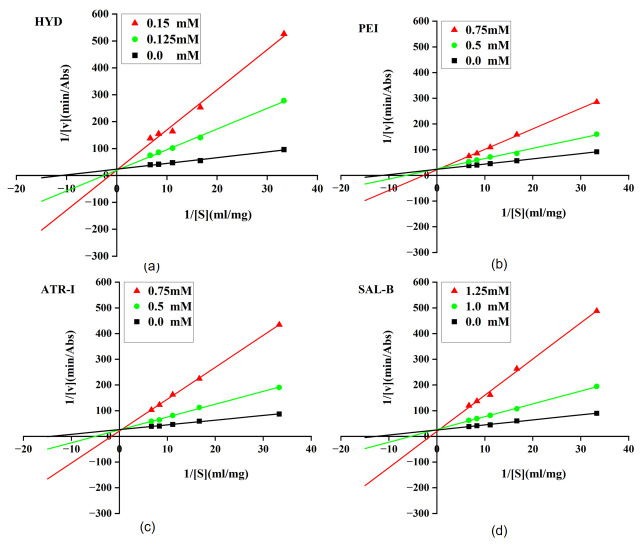
Lineweaver–Burk plots for PL inhibition by four compounds (**a**) HYD; (**b**) PEI; (**c**) ATR-I and (**d**) SAL-B.

The inhibitory activities of the six compounds were evaluated via an in vitro enzyme assay. The positive control, **Orlistat**, exhibited an IC_50_ value of 0.481 ± 0.023 μM. For comparative purposes, we assessed the inhibitory activity of **quercetin**, a widely investigated natural product inhibitor of PL. The IC_50_ obtained for quercetin was 0.231 ± 0.034 mM, consistent with the literature values ranging from 10 μM to 421.1 μM [54]. For the candidate compounds, the IC_50_ values were determined as follows: **HYD**, 0.128 ± 0.009 mM; **ATR-I**, 0.584 ± 0.031 mM; **PEI**, 0.748 ± 0.042 mM; **SAL-B**, 1.147 ± 0.065 mM; and **MUL-A**, 13.410 ± 0.724 mM. **LIN** showed no detectable inhibitory activity at concentrations up to 50 mM. Subsequently, for the four compounds (**HYD**, **ATR-I**, **PEI**, and **SAL-B**) with relative high activity, their inhibition kinetics were performed by measuring enzyme activity at fixed inhibitor concentrations and varying substrate concentrations. The resulting Lineweaver–Burk plots (Figure 6) demonstrated that the slopes of the fitted lines increased progressively with rising inhibitor concentration, while the *y*-axis intercepts remained essentially unchanged. These kinetic features indicated that all four compounds act as competitive inhibitors of PL.

## 4. Discussion

In the present study, we combined in silico screening and in vitro enzymatic assays to systematically identify potential PL inhibitors from TCM-derived compounds, with key results summarized in Table 3. The six compounds with docking scores ranging from −9.9 to −9.0 kcal/mol were chosen for further study. Molecular dynamics (MD) trajectory analysis showed that the ligand-bound PL maintained good structural stability with mean RMSD within 0.212 nm. In contrast, the mean RMSD values of the compounds ranged from 0.028 to 0.389 nm, indicating evident differences in their binding stability. MM/PBSA calculations showed that the binding affinity of the candidates were in the range from −21.24 ± 0.39 to −12.03 ± 0.40 kcal/mol. In vitro enzymatic analysis demonstrated that five of the six selected compounds displayed inhibitory activity against PL, with IC_50_ values ranging from 0.128 ± 0.009 mM (**HYD**) to 13.410 ± 0.724 mM (**MUL-A**). These IC_50_ values were comparable to that of positive control of **quercetin** (0.231 ± 0.034 mM), but significantly higher than that of the positive control **orlistat** (IC_50_ = 0.481 ± 0.023 μM, *p* < 0.05). This is because **orlistat** binds to PL via covalent interactions, whereas most natural products interact with PL primarily through non-covalent forces, thus generally exhibiting weaker inhibitory activity against PL than **orlistat**.

Although MM/PBSA can provide a rapid estimation of relative binding affinities, it is well documented in the literature that this method is not suitable for quantitatively reproducing experimental IC_50_ values, due to approximations in solvation models, insufficient entropy estimation, and the use of end-point averaging rather than rigorous free energy integration [29]. In our current case, **PEI** showed the most favorable predicted binding affinity, with a binding free energy of −21.24 ± 0.39 kcal/mol, but displayed relatively weak inhibitory activity (IC_50_ = 0.748 ± 0.042 mM). In contrast, **HYD** exhibited a moderate binding free energy (−15.58 ± 0.85 kcal/mol) yet the strongest in vitro potency (IC_50_ = 0.128 ± 0.009 mM). One plausible explanation for this discrepancy is that the entropic penalty was neglected in the MM/PBSA calculations, which may lead to substantial overestimation of the binding affinities of flexible ligands [29]. A prior study reported that each rotatable bond in flexible ligands incurs an entropic penalty of roughly 0.47 kcal/mol upon binding to target proteins [55]. In the present study, **SAL-B**, **MUL-A**, and **LIN** exhibited 14, eight, and seven rotatable bonds, respectively, conferring considerable structural flexibility. This feature corresponded to higher mean RMSD values throughout the simulations (Table 3), thereby leading to an overestimation of their binding affinities by 3–7 kcal/mol. In contrast, **HYD**, **ATR-I**, and **PEI** are structurally rigid, with no more than two rotatable bonds; consequently, entropic contributions exert only a negligible influence on their calculated binding energies. Consistent with these computational findings, in vitro experimental results further validated that **HYD**, **ATR-I**, and **PEI** possessed superior lipase inhibitory activities compared with the three conformationally flexible molecules above.

In the present study, **HYD** exhibited the most potent inhibitory activity against porcine pancreatic lipase (PL), with an IC_50_ value of 0.128 ± 0.009 mM (128 μM). Structurally, **HYD** is a luteolin derivative generated by the methylation of the 7-OH on the A-ring of **luteolin**. Previous studies have demonstrated that the free hydroxyl groups on the A- and B-rings of flavones are essential for their PL inhibitory activity [34], while methylation [56,57] or glycosylation [57,58] of these hydroxyl moieties can significantly attenuate such activity. For example, methylation of the 4′-hydroxyl group of **luteolin** increased its IC_50_ from 259 μM to 3033 μM for the methylated product **hesperetin** [57]. Consistently, luteolin has been reported to inhibit PL with a lower IC_50_ of 99 μM [57], showing stronger potency than **HYD**, indicating that methylation of the 7-hydroxyl group in luteolin impairs its PL inhibitory activity. This structure–activity relationship (SAR) also provides a clear explanation for the negligible PL inhibitory activity of linarin (**LIN**), a flavone glycoside substituted with 5-OH, 7-O-rutinoside, and 4′-OMe. **LIN** can be regarded as a derivative of apigenin (5,7,4′-OH), modified by 4′-O-methylation on the B-ring and 7-O-glycosylation on the A-ring. In contrast, **apigenin** has been reported to inhibit PL with an IC_50_ of 256 μM [56]. For **LIN**, however, the loss of free hydroxyl groups caused by 4′-O-methylation may impair its capacity to form hydrogen bonds, while 7-O-glycosylation introduces a bulky hydrophilic moiety. Together, these modifications render it unable to form a favorable interaction with PL, as reflected by its vibrant fluctuations in RMSD during the MD simulation (Figure 2c), thus leading to the nearly complete abolition of PL inhibitory activity.

Several limitations of the present study should be acknowledged. First, porcine pancreatic lipase was used in the inhibitory assays instead of human pancreatic lipase. Although porcine lipase is widely accepted as a reliable surrogate for the preliminary screening of lipase inhibitors [59,60] owing to its high structural and functional homology with human lipase [53], subtle differences in inhibitor sensitivity between species still cannot be ruled out [49,61]. Accordingly, the physiological relevance of our findings to humans needs to be further validated using human-derived pancreatic lipase in future studies. Second, cytotoxicity evaluations were not performed in the current work. Since cytotoxicity represents a critical prerequisite for assessing the safety and druggability of bioactive compounds, the lack of such data limits a comprehensive evaluation of the potential of the tested compounds for further therapeutic development. Finally, although the screened compounds exhibited inhibitory activity against pancreatic lipase, their inhibitory potency was relatively modest, particularly for **SAL-B** and **MUL-A**, primarily due to their high hydrophilicity arising from the presence of multiple hydroxyl groups. Therefore, further rational structural modification and optimization will be required to moderately reduce their polarity (e.g., via glycosidic bond hydrolysis or hydroxyl methylation), thereby enhancing their binding to the hydrophobic pocket of pancreatic lipase and meeting the criteria for potential practical applications.

## 5. Conclusions

In conclusion, this study demonstrates the utility of integrating in silico and in vitro approaches for identifying natural pancreatic lipase (PL) inhibitors from traditional Chinese medicines (TCMs). This integrated approach not only improved the efficiency and accuracy of screening potential PL inhibitors but also revealed the dynamic binding characteristics of ligand–PL complexes. We identified five TCM-derived compounds (**HYD**, **ATR-I**, **PEI**, **SAL-B**, and **MUL-A**) that exhibit PL inhibitory activity. Notably, **HYD**, **ATR-I**, and **PEI** were confirmed as effective inhibitors with IC_50_ values of 0.128 ± 0.009, 0.584 ± 0.031, and 0.748 ± 0.042 mM, respectively, which have not been previously reported to inhibit PL. This finding expands the pool of natural PL inhibitors and provides new hit compounds for anti-obesity drug development. Future in vivo studies and rational structural modification (e.g., moderate polarity reduction to enhance binding to the PL hydrophobic pocket) will facilitate the translation of these findings into practical applications. This work validates the feasibility of the integrated screening strategy and lays a solid foundation for subsequent research on natural anti-obesity agents.

## Figures and Tables

**Table 1:** Docking results of the selected candidates of PL inhibitors.

Mol_ID	Mol_Name	Score	Hydrogen Bonds	Hydrophobic Interactions
MOL000043	Atractylenolide I (ATR-I)	−9.0	Ser152, His263	Phe77, Tyr114, Ala178, Phe215
MOL001790	Linarin (LIN)	−9.3	Cys181, Glu183	Phe182, Thr185, Val210, Leu213
MOL005530	Hydroxygenkwanin (HYD)	−9.3	Gly76, Phe77, His151	Arg256, Tyr114, Ala260, Leu264
MOL007074	Salvianolic Acid B (SAL-B)	−9.3	Asp79	Ala259, Ile78, Tyr114, Phe215, Arg256
MOL004451	Peiminine (PEI)	−9.4	None	Phe77, Ile78, His151, Trp252, Thr255, Arg256, Ala259, Leu264
MOL012687	Mulberroside A (MUL-A)	−9.9	Gly76, Thr255, Arg256	Phe77, ILE78, Tyr114, Pro180, Ile209, Phe215, Ala259, Leu264

**Table 2:** Binding free energies (kcal/mol) of PL with the six compounds calculated by MM/PBSA.

Compound	ΔE_VDW_	ΔE_ELE_	ΔE_PB_	ΔE_NPOLAR_	ΔG_GAS_	ΔG_SOLV_	ΔG_TOTAL_
**ATR-I**	−17.08 ± 0.51	−5.35 ± 0.50	11.09 ± 0.60	−2.11 ± 0.04	−22.43 ± 0.90	8.99 ± 0.56	−13.44 ± 0.45
**LIN**	−26.47 ± 0.72	−43.49 ± 1.61	53.71 ± 1.39	−3.29 ± 0.05	−69.96 ± 1.62	50.42 ± 1.37	−19.54 ± 0.43
**HYD**	−35.35 ± 0.39	−6.88 ± 0.60	29.99 ± 0.93	−3.34 ± 0.02	−42.23 ± 0.82	26.65 ± 0.92	−15.58 ± 0.85
**SAL-B**	−24.91 ± 0.53	75.58 ± 2.13	−59.06 ± 1.88	−3.63 ± 0.03	50.66 ± 2.04	−62.70 ± 1.87	−12.03 ± 0.40
**PEI**	−38.97 ± 0.29	−13.26 ± 0.65	34.85 ± 0.50	−3.87 ± 0.04	−52.23 ± 0.62	30.99 ± 0.50	−21.24 ± 0.39
**MUL-A**	−48.58 ± 0.43	−28.76 ± 0.76	68.96 ± 1.07	−4.95 ± 0.03	−77.34 ± 0.84	64.01 ± 1.06	−13.33 ± 0.58

**Table 3:** Summary of the key results related to the activity of the six selected compounds.

Compound	Docking Score (kcal/mol)	Mean RMSD (nm)		ΔG_TOTAL_ (kcal/mol)	IC_50_ (mM)	Inhibition
		Protein	Compound			Type
**ATR-I**	−9.0	0.202	0.028	−13.44 ± 0.45	0.584 ± 0.031	Competitive
**LIN**	−9.3	0.185	0.256	−19.54 ± 0.43	>50.0	N.D.
**HYD**	−9.3	0.212	0.114	−15.58 ± 0.85	0.128 ± 0.009	Competitive
**SAL-B**	−9.3	0.196	0.389	−12.03 ± 0.40	1.147 ± 0.065	Competitive
**PEI**	−9.4	0.202	0.144	−21.24 ± 0.39	0.748 ± 0.042	Competitive
**MUL-A**	−9.9	0.212	0.221	−13.33 ± 0.58	13.410 ± 0.724	N.D.

N.D. = Not determined.

## Data Availability

The original contributions presented in this study are included in the article/Supplementary Material. Further inquiries can be directed to the corresponding authors.

## Supplementary Materials

The following supporting information can be downloaded at: https://www.mdpi.com/article/10.3390/cimb48040404/s1.

## Abbreviations

The following abbreviations are used in this manuscript:

PL	Pancreatic lipase
TCM	Traditional Chinese medicine
MD	Molecular dynamics
MM/PBSA	Mechanics/Poisson-Boltzmann surface area
RMSD	Root Mean Square Deviation
Rg	Radius of Gyration
pNPP	p-nitrophenyl palmitate
ATR-I	Atractylenolide I
LIN	Linarin
HYD	Hydroxygenkwanin
SAL-B	Salvianolic Acid B
PEI	Peiminine
MUL-A	Mulberroside A
