# Supplementary material for: Identification of Potential Pancreatic Lipase Inhibitors from Traditional Chinese Medicines via Molecular Docking, Molecular Dynamics Simulation and In Vitro Validation
Source: Curr Issues Mol Biol. 2026 Apr 15;48(4):404. doi: 10.3390/cimb48040404 (PMC13114888; doi:10.3390/cimb48040404)
Supplement: Supplementary file 1 [file cimb-48-00404-s001.zip › cimb-4190767-supplementary.pdf]

Table S1. Autodock Vina-based docking scores of Traditional Chinese Medicine derived pancreatic lipase inhibitors

MOL-ID	Name	IC_{50} (uM)	Docking Score (kcal/mo)	Reference
MOL002008	myricetin	335.7	-8.8	3
MOL007930	hesperidin	52.4	-10.2	3
MOL002051	isoquercitrin	25	-7.0	3
MOL000737	morin	20.56	-8.0	5
MOL007476	ginsenoside rb1	17.6	-6.9	2
MOL013296	fustin	13.7	-7.5	3
MOL011550	sciadopitysin	12.78	-9.0	4
MOL000739	morusin	9.94	-8.5	5
MOL000006	luteolin	7.1	-9.2	3
MOL002509	ginkgetin	6.9	-9.3	4
MOL012695	kuwanon G	4.85	-6.9	5
MOL000738	kuwanon C	4.47	-7.0	5
MOL004401	bilobetin	3.57	-9.0	4
MOL012750	sanggenone C	3.0	-7.6	5
MOL002511	isoginkgetin	2.9	-9.6	4
MOL012334	ginsenoside Rg3	2.47	-7.9	2
MOL002067	hypericin	0.95	-7.5	1

References

1. Hou XD, Qin XY, Hou J, Tang H, Ge GB. The potential of natural sources for pancreatic lipase inhibitors: a solution of the obesity crisis? Expert Opin Drug Discov. 2022,17(12):1295-1298.

2. Wang AQ, Wang YJ, Zhang J, Fan YM, Li SY, Zou LW. Comparative study on inhibitory effects of ginsenosides on human pancreatic lipase and porcine pancreatic lipase: structure-activity relationships and inhibitory mechanism. Nat Prod Res. 2024,38(12):2031-2039.

3. Buchholz T, Melzig MF. Polyphenolic Compounds as Pancreatic Lipase Inhibitors. Planta Med. 2015,81(10):771-83.

4. Liu PK, Weng ZM, Ge GB, Li HL, Ding LL, Dai ZR, Hou XD, Leng YH, Yu Y, Hou J. Biflavones from Ginkgo biloba as novel pancreatic lipase inhibitors: Inhibition potentials and mechanism. Int J Biol Macromol. 2018,118(Pt B):2216-2223.

5. Hou XD, Ge GB, Weng ZM, Dai ZR, Leng YH, Ding LL, Jin LL, Yu Y, Cao YF, Hou J. Natural constituents from Cortex Mori Radicis as new pancreatic lipase inhibitors. Bioorg Chem. 2018, 80:577-584.

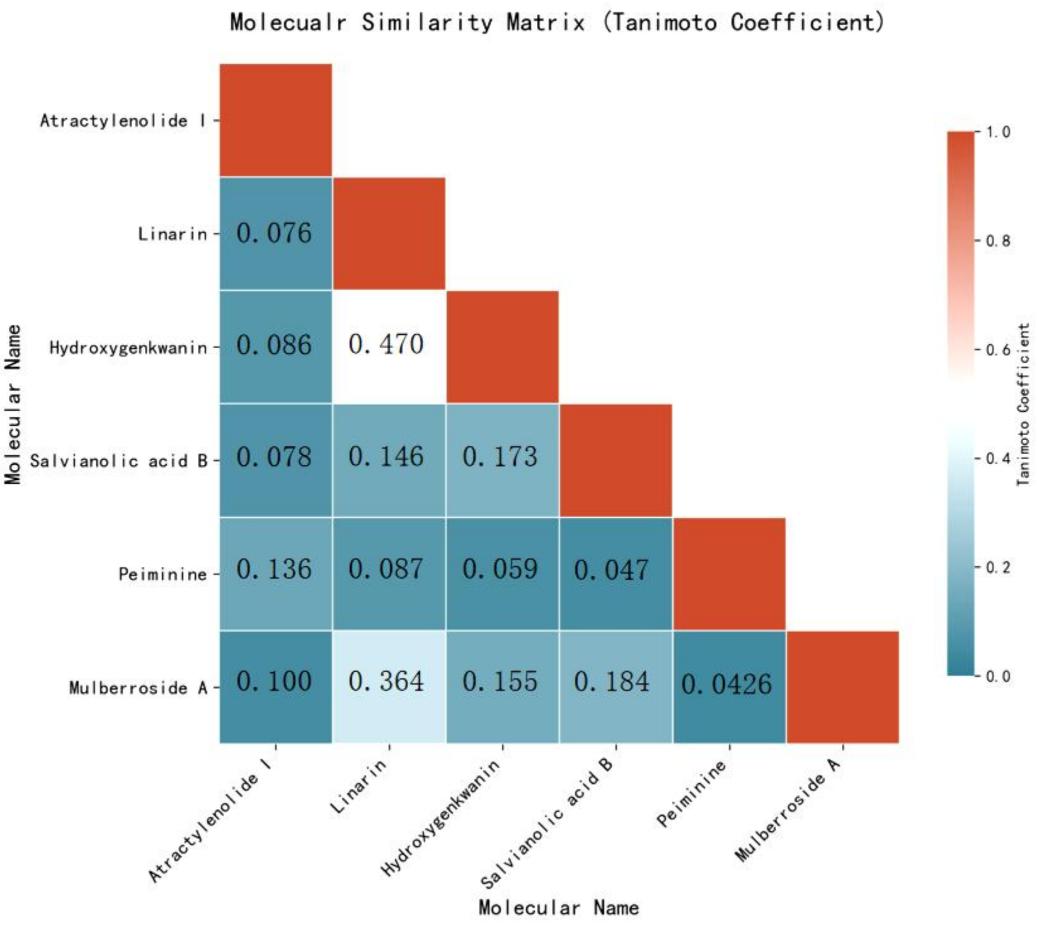

Figure S1. Hotmap of similarity matrix of the selected compounds.
